# Genome-wide estimates of genetic diversity, inbreeding and effective size of experimental and commercial rainbow trout lines undergoing selective breeding

**DOI:** 10.1186/s12711-019-0468-4

**Published:** 2019-06-06

**Authors:** Jonathan D’Ambrosio, Florence Phocas, Pierrick Haffray, Anastasia Bestin, Sophie Brard-Fudulea, Charles Poncet, Edwige Quillet, Nicolas Dechamp, Clémence Fraslin, Mathieu Charles, Mathilde Dupont-Nivet

**Affiliations:** 1 GABI, INRA, AgroParisTech, Université Paris-Saclay, 78350 Jouy-en-Josas, France; 2 SYSAAF Section Aquacole, Campus de Beaulieu, 35000 Rennes, France; 3 SYSAAF Section Avicole, Centre INRA Val de Loire, 37380 Nouzilly, France; 4 GDEC, INRA, Université Clermont-Auvergne, 63039 Clermont-Ferrand, France

## Abstract

**Background:**

Selective breeding is a relatively recent practice in aquaculture species compared to terrestrial livestock. Nevertheless, the genetic variability of farmed salmonid lines, which have been selected for several generations, should be assessed. Indeed, a significant decrease in genetic variability due to high selection intensity could have occurred, potentially jeopardizing the long-term genetic progress as well as the adaptive capacities of populations facing change(s) in the environment. Thus, it is important to evaluate the impact of selection practices on genetic diversity to limit future inbreeding. The current study presents an analysis of genetic diversity within and between six French rainbow trout (*Oncorhynchus mykiss*) experimental or commercial lines based on a medium-density single nucleotide polymorphism (SNP) chip and various molecular genetic indicators: fixation index (*F*_ST_), linkage disequilibrium (LD), effective population size (*N*_*e*_) and inbreeding coefficient derived from runs of homozygosity (ROH).

**Results:**

Our results showed a moderate level of genetic differentiation between selected lines (*F*_ST_ ranging from 0.08 to 0.15). LD declined rapidly over the first 100 kb, but then remained quite high at long distances, leading to low estimates of *N*_*e*_ in the last generation ranging from 24 to 68 depending on the line and methodology considered. These results were consistent with inbreeding estimates that varied from 10.0% in an unselected experimental line to 19.5% in a commercial line, and which are clearly higher than corresponding estimates in ruminants or pigs. In addition, strong variations in LD and inbreeding were observed along the genome that may be due to differences in local rates of recombination or due to key genes that tended to have fixed favorable alleles for domestication or production.

**Conclusions:**

This is the first report on ROH for any aquaculture species. Inbreeding appeared to be moderate to high in the six French rainbow trout lines, due to founder effects at the start of the breeding programs, but also likely to sweepstakes reproductive success in addition to selection for the selected lines. Efficient management of inbreeding is a major goal in breeding programs to ensure that populations can adapt to future breeding objectives and SNP information can be used to manage the rate at which inbreeding builds up in the fish genome.

**Electronic supplementary material:**

The online version of this article (10.1186/s12711-019-0468-4) contains supplementary material, which is available to authorized users.

## Background

Rainbow trout (*Oncorhynchus mykiss*) is native to the Pacific drainages of North America and also to Kamchatka in Russia. This fish was introduced at the end of the nineteenth century to waters on all continents except Antarctica, for recreational angling and aquaculture purposes. Rainbow trout is one of the main species of fish reared in cold freshwater around the world, particularly in Europe, North America and Chile [1]. For several decades, the rainbow trout farming industry has been endeavoring to continually increase production efficiency and sales by increasing rearing densities, improving diets, water quality and recirculation technology, controlling sexual maturation and gender, or developing genetically superior lines of fish for improved growth, fillet quality and disease resistance. Recent access to the genome sequence of rainbow trout [2], genetic maps [3–5], and a medium-throughput genotyping chip [6] of 57,501 single nucleotide polymorphisms (SNPs) offer new perspectives for research and organization of trout breeding programs, which may be more effective than those that are historically based on phenotypic or genealogical selection of the broodstock.

Selective breeding can contribute to a significant decrease in the genetic variability of farmed populations, jeopardizing long-term genetic progress as well as reducing the adaptive capacities of populations in the event of a change in the environment [7]. Greater genetic variability within a population increases the likelihood that some of its individuals will have alleles that are better adapted to environmental fluctuations and are likely to survive and to transmit to their offspring alleles and favorable genetic characteristics.

Selection, mutation, migration between populations and genetic drift constitute the different evolutionary forces that can create linkage disequilibrium (LD), e.g. preferential association of alleles at different loci. The analysis of LD plays a central role in many areas of population genetics, including: the determination of genetic maps, ascertainment of levels of recombination at the population level, and estimation of effective population sizes (*N*_*e*_). The *N*_*e*_ of a population is a concept that was developed by Wright [8] and defined as the size of an idealized population undergoing the same rate of genetic drift as the population under study. A number of methods to estimate *N*_*e*_ from demographic, pedigree, or molecular data have been proposed (e.g. Leroy et al. [9]). Most of the molecular estimates are derived from LD or temporal methods that give indirect estimators of *N*_*e*_ [10] through the use of a genetic index: the squared correlation *r*^2^ of alleles at different gene loci using a single sample in the LD method or the standardized variance in allele frequency between two temporal samples in the temporal approach. The temporal method is based on the theory that *N*_*e*_ is the only parameter that is needed to determine rates of change in allele frequency at neutral loci in a population in Hardy–Weinberg equilibrium [11]. The LD method of estimating *N*_*e*_, developed by Sved [12] and modified by Hill [13], is based on the principle that in closed finite populations in approximate drift–mutation–recombination equilibrium and constant census size, associations between alleles at different neutral loci are a function of the population’s *N*_*e*_. Therefore, if an estimate of the rate of recombination between loci is available, *Ne* can be derived from the expected level of allele association across loci $E\left( {r^{2} } \right)$.

The maintenance of genetic diversity within a population is achieved by maximizing *N*_*e*_, or equivalently, by minimizing the increase in inbreeding across generations. A molecular estimate of the inbreeding coefficient can be based on measuring long stretches of consecutive homozygous genotypes in each individual, the so-called runs of homozygosity (ROH; McQuillan et al. [14]). Long homozygous regions throughout the genome result from mating between close relatives, reduction in population size, and selection. Thus, population structure and selection effects can be assessed based on the distribution and location of ROH. Several studies have shown that characterizing inbreeding based on ROH provides a better measure of individual autozygosity because parents transmit identical haplotypes to their offspring than estimating overall inbreeding based on pedigree information, because kinships between base animals are not accounted for in pedigree files [15, 16].

In the present study, we used medium-density SNP chips to analyze the genetic diversity within and between six lines of rainbow trout. The objective of this research was to evaluate the impact of selection practices on genetic diversity through basic population indices and individual molecular genetics statistics. Our results may help to evaluate breeding practices in the light of the genetic evolution of farmed fish populations under selection. In addition, it provides commercial breeders a better understanding of the genetic composition of their selected lines and allows them to design and implement effective genomic breeding programs.

## Methods

### Background of the selected lines

**Fig. 1:**
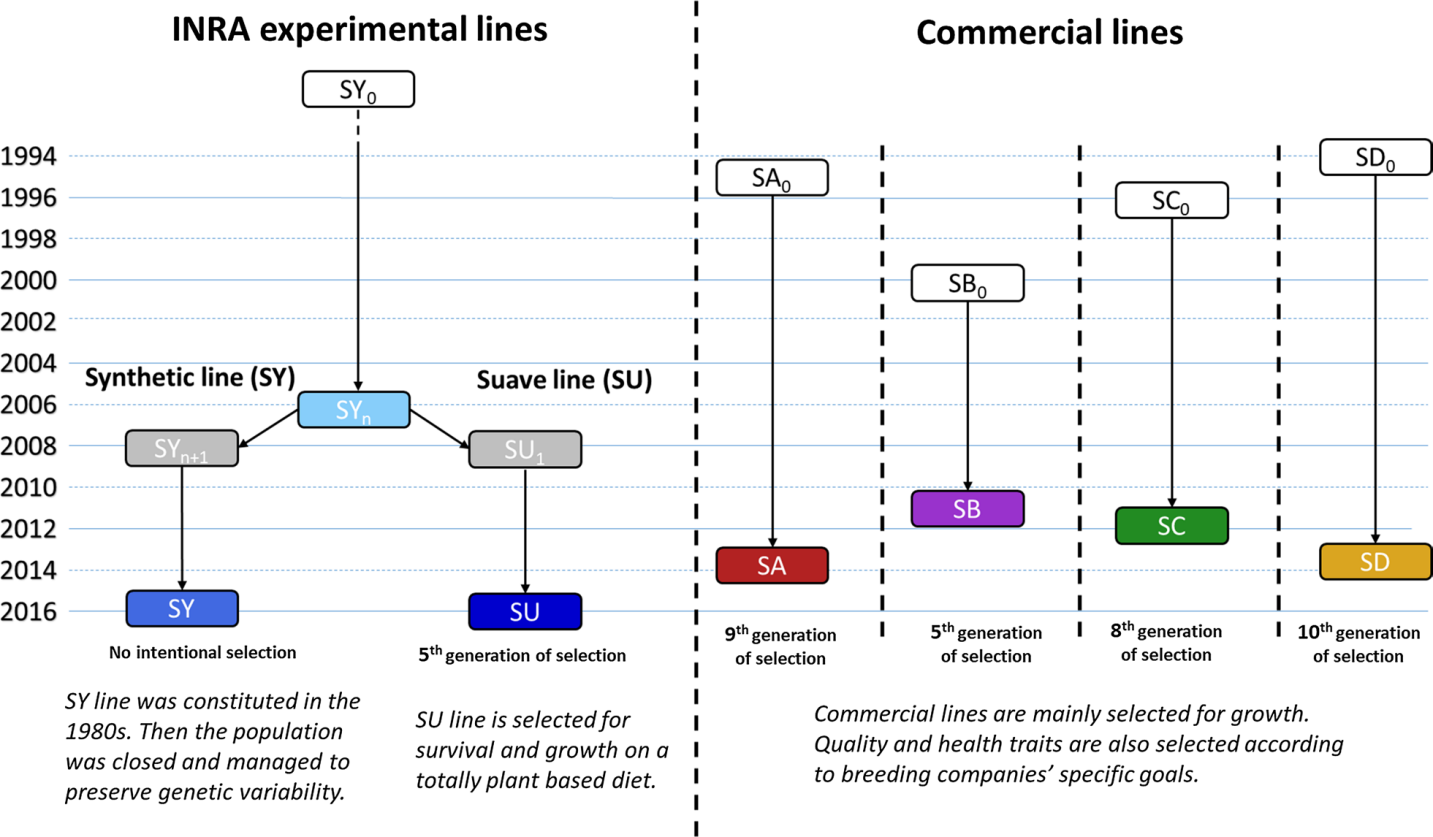
Chronology of the breeding schemes that were set up for the six French rainbow trout lines

The INRA synthetic line was initially developed by intercrossing several domesticated lines of rainbow trout in order to create a population with a large genetic variability. The line was constituted from a mixture of French farmed populations with some new introductions from Denmark and USA in the early 1980s. The population was then closed to outside germplasm, and has since been bred without any intentional selection in order to maintain genetic diversity using a full factorial mating design between 2-year-old breeding animals (about 60 dams and 80 sires, each year). Individuals genotyped for the current study were from the 2006 birth year class (SY_n_), and from the 2016 birth year class (SY) (Fig. 1).

In early 2008, the first generation of the Suave (SU_1_) line was spawned from a full factorial cross among SY_n_ founder parents (32 dams and 44 sires). The SU line in the current study is the 5th generation of selection for survival and growth on a totally plant-based diet provided from first feeding [17]. Each generation of the SU line is created from a full factorial design between at least 40 dams and 45 sires. The selection is a sequential phenotypic selection: among the surviving animals, fish with the longest body length are selected through three to four selection events during the first year. The proportion of selected fish at each generation is around 4 to 5%. After the first three generations of selection, survival of SU fish was increased by 15% compared to SY fish and their final weight (at 197 days post-fertilization) was increased by 48% due to a 19% increase in feed intake when fish were fed a plant-based diet [18].

Breeding schemes for the four commercial lines (SA, SB, SC and SD) have been based on closed populations that were selected for at least five generations at the time the cohorts were sampled for the study. The samples represented the 5th, 8th, 9th and 10th generation of selection in SB, SC, SA and SD breeding companies, respectively (Fig. 1). The history and the composition of these lines before the initiation of their structured selection program are unknown, however all four lines were created using sex-reversed males from INRA.

The four commercial lines are reproduced by an artificial fertilization protocol to balance the contribution of each parent by partial factorial mating of 6 to 10 dams and 10 sires according to the PROSPER procedure [19]. Each spawn is subdivided in 10 subgroups, with each subgroup being fertilized by a different sire before recombining the dam subgroups. Each of the 10 sires is used to fertilize subgroups from 6 to 10 different dams. At eyed stage, similar numbers of eggs from each dam are mixed together to balance the maternal contributions in the fry rearing tank (effectively also balancing the contribution of each sire). This procedure is expected to minimize inbreeding, since a very large number of families per generation are then created (> 600) limiting the risk of mating related fish from the same full-or half-sib families [20].

Commercial lines are selected for growth traits (mainly weight and length at 18 months) by optimized within-group mass selection and a 3 to 10% selection pressure according to the PROSPER procedure [19]. To maintain the four commercial lines, a minimum number of broodstock (> 150) is selected at each generation. In addition, for the SA, SB and SC lines, a sib-based selection is performed based on carcass quality traits (carcass and fillet yields) assisted by ultrasound prediction [21, 22]. The management of inbreeding within these three lines has been based on DNA parentage assignment [23], and has since been improved over the last 10 years by using an optimal pedigree-based selection strategy [24].

### Genotypes

The 57,501 SNPs (57K SNP) Axiom^®^ Trout Genotyping array [6] was used to genotype 302 females from six French lines of rainbow trout at the INRA genotyping Platform Gentyane. Animals were sampled to represent the genetic diversity of their birth cohorts by avoiding full-sib relationships for the three commercial lines with pedigree information available (SA, SB, SC). Among the genotyped animals, eight individuals with more than 45% identity-by-state (IBS) with another individual were removed from the INRA and SD populations for which pedigrees are unknown. In addition, four animals with less than 95% of the SNPs genotyped were removed from the study. After editing, 290 genotyped animals were considered in the analysis, including 48, 48, 49, 48, 32, 32 and 33 fish from SA, SB, SC, SD, SU, SY_n_ and SY lines, respectively.

#### Subset of the 57K SNPs validated for the study

Quality control of SNPs was performed in several steps. First, all 57,501 SNP probes were positioned with a BLASTn^®^ procedure on the second genome assembly at the chromosome level Omyk_1.0 [25, 26] and only 50,820 SNPs with a unique position were retained. Although *Oncorhynchus mykiss* is a diploid species, there is still residual tetraploidy in the 2.2 Gb of the rainbow trout genome due to the fourth round salmonid whole-genome duplication event that occurred approximatively 96 million years ago [2, 27]. In addition, intraspecific variation in diploid chromosome number (2n varying between 58 and 64) exists in rainbow trout [28]. The American reference genome is based on a set of 29 pairs of chromosomes. However, all six French lines are expected to carry a set of 30 pairs of chromosomes, the American Omy25 being split into two chromosomes (French Omy25a and Omy25b).

Second, we used the Axiom Analysis Suite 2.0 software [29] to control the quality of the remaining markers on a large dataset of 3418 genotyped individuals from the six French rainbow trout lines (including the 302 individuals of the current study). Edits consisted in discarding 7711 SNPs with probe polymorphism or a call rate lower than 97%; 2995 SNPs for which no homozygous individual was observed for the minor allele on the full genotyped set; and 1689 SNPs that were monomorphic in all lines.

Third, regarding the genotypes for the remaining 38,425 SNPs, we performed a final quality control using PLINK v1.9 software [30] on each line set considered in the current study. We discarded SNPs with a very significant deviation from Hardy–Weinberg equilibrium (p value < 0.0001) in one or several populations. Thus, only 38,350 SNPs remained for the analysis of genetic diversity between populations (PCA and *F*_ST_ analysis).

Finally, we only retained the SNPs that had a sufficiently high minor allele frequency (MAF) to obtain reasonable results for each of the analyses. For the ROH studies (MAF ≥ 1%), 34,077–37,340 SNPs were kept depending on the lines. For LD calculation (MAF ≥ 5%), 31,190–34,723 SNPs were considered depending on the lines (see Additional file 1: Table S1).

#### Genetic structure of the population

Observed heterozygosity (Ho) and expected heterozygosity (He) for a population under Hardy–Weinberg equilibrium were derived with the PLINK v1.9 software [30]. Levels of genetic variation in the different lines were compared using a Kruskal–Wallis non-parametric test on Ho values. Genetic differentiation between populations was measured with pairwise *F*_ST_ estimates [31], using the VCFtools v0.1.13 software [32]. In addition, a principal component analysis (PCA) was performed with the R package Adegenet [33] to visualize the genetic structure of the lines.

### Linkage disequilibrium

To estimate LD, we used the squared correlation based on genotypic allele counts (number of non-reference alleles at each locus) using the PLINK v1.9 software [30]. This *r*^2^ value does not necessitate phasing, it is very similar to but not identical to the *r*^2^ estimate derived from haplotype frequencies [34].

Pairwise LD between adjacent SNPs and pairwise LD between all SNPs in a 60-Mb long window were derived for each chromosome and line. Mean *r*^2^ values were calculated for each chromosome and line by considering the following average distances between SNPs: 10 kb with a 20 kb-window; 50 kb and 100 kb with a 50 kb-window; 1, 3, 5, 10 and 30 Mb within a 100-kb window for each distance.

### Estimates of effective population size *N*_*e*_

Two metrics were considered to estimate *N*_*e*_: ($N_{{e_{t} }}$) was based on LD and could be derived for all populations, whereas ($N_{{e_{F} }}$) was based on temporal changes in allele frequency and could therefore only be calculated for the SY and SU lines.

For all populations, *N*_*e*_ was derived using the formula proposed by Sved [12] and based on the expected LD: $$E\left( {r^{2} } \right) \approx \frac{1}{{4cN_{e} + 1}}$$ in which *c* is the distance in Morgan between SNPs. Estimating *r*^2^ by sampling from the populations produces another source of error [13]; consequently, for sample size $$S$$, the formula is $$E\left( {r^{2} } \right) \approx \frac{1}{{1 + 4cN_{e} }} + \frac{1}{S}$$, which can be rearranged as: $$N_{e} \approx \frac{{1 + {\raise0.7ex\hbox{$1$} \!\mathord{\left/ {\vphantom {1 S}}\right.\kern-0pt} \!\lower0.7ex\hbox{$S$}} - E\left( {r^{2} } \right)}}{{4c\left[ {E\left( {r^{2} } \right) - {\raise0.7ex\hbox{$1$} \!\mathord{\left/ {\vphantom {1 S}}\right.\kern-0pt} \!\lower0.7ex\hbox{$S$}}} \right]}}$$.

*N*_*e*_ was estimated by transforming physical distance between SNPs into genetic distance *c*, based on the genetic map recently established for a French rainbow trout population: 10 cM corresponded to six Mb on average across the 30 chromosomes [5].

We derived *N*_*e*_ at *t* past generations ($N_{{e_{t} }}$) based on the equation $t = 1/2c$ based on the coalescent theory and the assumptions of Wright–Fisher model [35], considering $E\left( {r^{2} } \right)$ as the mean *r*^2^ across all the chromosomes and all SNP pairs across a distance of *c*.

To derive mean *r*^2^, the window around *c* was determined for any *t* generation by considering the interval ]*t *− 0.5; t + 0.5]; for instance at $t = 1$, *c* averaged 33.3 Mb between SNPs with possible values ranging from 20 Mb ($t = 1.5$) to 60 Mb ($t = 0.5$). For all populations, $N_{{e_{t} }}$ was calculated up to the 10th generation back. Standard errors for these *N*_*e*_ estimates were derived according to equations (5) and (6) of Hill [13] considering the chromosome average ${\text{V}}\left( {r^{2} } \right)$ over all SNP pairs across a distance of *c* per chromosome.

Second, *N*_*e*_ was derived by considering the temporal approach [11] and the formula proposed by Nei and Tajima [36]: $N_{{e_{F} }} = \frac{t}{{2 {\hat{F}_{k} - 1/S_{0} - {1/S_{t} }}}}$, with $\hat{F}_{k}$ the standardized variance of allele frequency (corresponding to the Wright inbreeding coefficient), $S_{0}$ and $S_{t}$ sample sizes at generation 0 and $t$, respectively.

$\hat{F}_{k}$ was derived as $\hat{F}_{k} = 2*\left( {x - y} \right)^{2} *\left[ {\frac{1}{x + y} + \frac{1}{2 - x - y}} \right]$ as proposed by Pollak [37] and considering only two alleles per SNP, where $x$ is the major allele frequency at generation 0 and *y* is the allele frequency at generation *t*. Then, $\hat{F}_{k}$ was averaged for all SNPs to estimate $N_{{e_{F} }}$.

#### Runs of homozygosity

ROH were identified for each fish within all lines using PLINK v1.9 [30]. ROH were defined by sliding windows with a minimum length of one Mb containing at least 30 homozygous SNPs. The maximum gap between two consecutive homozygous SNPs in a run was set to the default value of one Mb. To ensure that the low SNP density did not falsify ROH length, a minimum density of one SNP every 100 kb was also set (the median and average distances between adjacent SNPs across all the genomes were 31 and 56 kb, respectively). No more than five SNPs with missing genotypes were allowed per window and up to one possible heterozygous genotype was permitted per ROH. These parameters are common practice when deriving ROHs in animal livestock populations with 2–3 Gb genome sizes and 50 K SNP chips. In most studies, the minimum number of SNPs to constitute a ROH is in-between 20 and 50 [38–40]. According to the formula proposed by Purfield et al. [15], the minimum number of SNPs that constituted a ROH should be about 35 SNPs in each line in order to minimize the number of ROH that may occur only by chance in the SNP panel (accepting a 5% false positive rate).

Mean number of ROH (N_ROH_), number of SNP per ROH (S_ROH_), length of ROH (L_ROH_) and percentage of ROH segment longer or equal to 10 Mb were calculated per individual and line. To identify the genomic regions most commonly associated with ROH, the percentage of individuals with a SNP in a ROH segment was calculated by counting the number of times the SNP was detected in a ROH within the population. This count was plotted against the position of the SNP along the chromosome.

### Estimates of total and recent inbreeding

Inbreeding coefficients (*F*_ROH_) were calculated as the sum of ROH lengths of an individual divided by the total length of the autosomal genome covered by SNPs. The total size of the autosomal genome covered by SNPs was calculated within each line and chromosome. Genome regions with a gap between two adjacent SNPs larger than 1 Mb (authorized gap to derive a ROH) were deducted from the total size of the genome covered by SNPs. In addition, recent inbreeding (*F*_ROH>10Mb_) was derived as the sum of the lengths of ROH segments longer than 10 Mb in order to estimate inbreeding occurring within the last three generations (10 Mb = 0.166*c* and $t = 1/2c$; therefore $t = 3$ generations for 10 Mb). Chromosome *F*_ROH_ was also derived as the sum of the lengths of ROH in a given chromosome of an individual divided by the total length of the chromosome genome covered by SNPs and then averaged for each line.

## Results

### Population genetic statistics and structure

When considering SNPs irrespective of their MAF, the observed heterozygosity (Ho) ranged from 33.5 to 35.3% (Table 1). On average, values for Ho went up to 36.5 to 38.0% when only SNPs with a MAF ≥ 5% within the population were considered (Table 1). Frequencies at almost all loci were in agreement with Hardy–Weinberg expectations in each line, although, on average across all the SNPs the expected heterozygosity (He) tended to be one point below that of Ho in all selected populations. Kruskal–Wallis tests showed that there was a very significant difference in Ho (p value < 0.00001) between the lines when considering all SNPs and still some significant variation (p value < 1%) when considering SNPs with a MAF ≥ 5%. Comparing the Ho values two-by-two showed that the commercial SA, SB and SC lines had significantly lower heterozygosity values than the SD and INRA experimental lines.

**Table 1:** Observed (Ho) and expected (He) heterozygosity for each rainbow trout line

Line	N	All SNPs		SNPs with a MAF ≥ 5%	
		Ho	He	Ho	He
SA	48	33.50*	32.47	36.54*	35.41
SB	48	34.24*	33.34	36.94	35.96
SC	49	33.92*	33.17	36.92	36.10
SD	48	35.01	34.18	37.47	36.58
SU	32	35.33	34.10	37.98	36.65
SY_n_	32	35.30	34.90	37.49	37.06
SY	33	35.23	34.87	37.47	37.05

* Significant p value (< 1%) for Kruskal–Wallis test for Ho value in comparison to Ho value for SY line

**Fig. 2:**
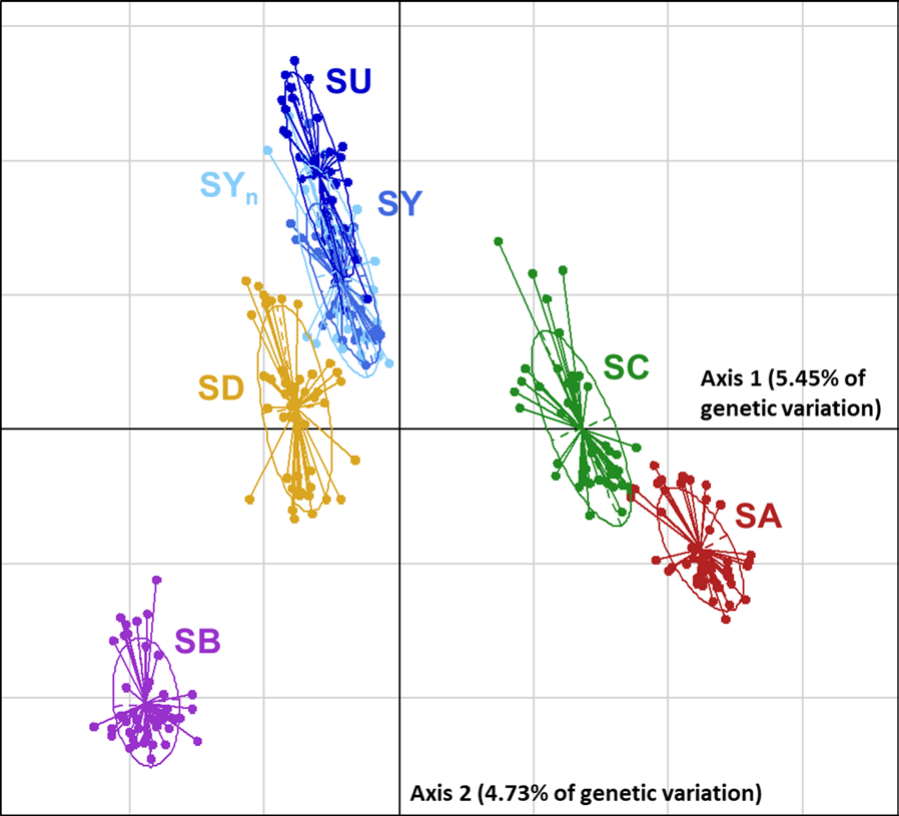
Principal component analysis plot of the genetic diversity between the French rainbow trout lines. PCA was performed with 290 individuals and 38,350 SNPs

Results showed moderate genetic differentiation between lines. Overall, 10% of the total genetic variation is explained by the first two PCA axes (Fig. 2). Except for the null differentiation between the two cohorts of the INRA unselected line (Table 2), *F*_ST_ values ranged from 0.02 (SY_n_–SU) to 0.15 (SA–SB). All commercial lines were moderately distant from each other with *F*_ST_ ranging from 0.09 to 0.15. The SD line was the commercial line that was genetically the closest to the INRA SY line, whereas the SA and SB were the most distant.

**Table 2:** Pairwise *F*_ST_ between lines of French rainbow trout

Line	SA	SB	SC	SD	SU	SY_n_
SB	0.150					
SC	0.115	0.139				
SD	0.113	0.092	0.101			
SU	0.128	0.128	0.113	0.08		
SY_n_	0.101	0.096	0.086	0.057	0.024	
SY	0.105	0.102	0.091	0.065	0.038	0.007

### Linkage disequilibrium analysis

**Fig. 3:**
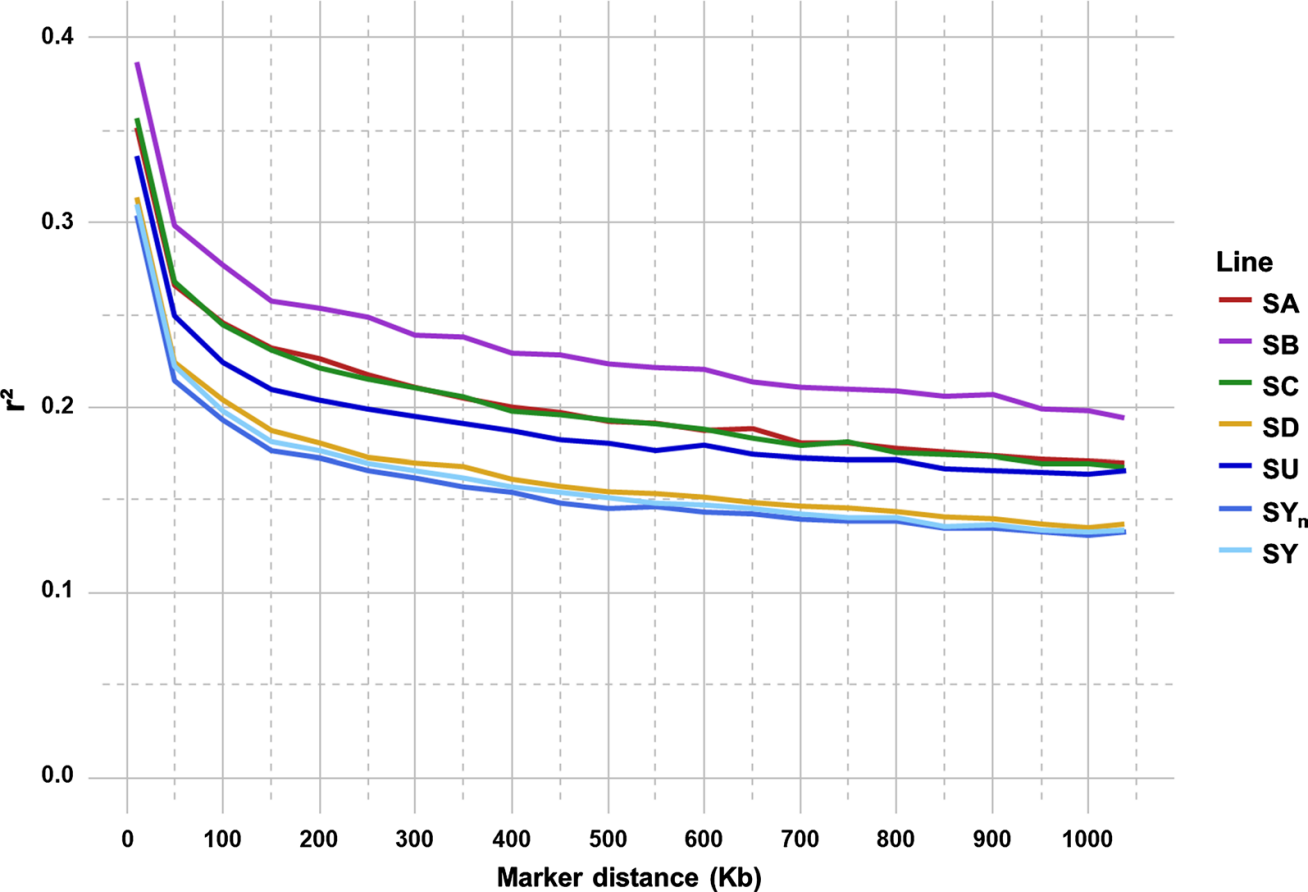
Linkage disequilibrium (*r*^2^) decay with physical distance between markers in each of the six French rainbow trout lines

As expected, average *r*^2^ tended to decrease with increasing distance between pairs of SNPs in all the populations studied, the most rapid decline being over the first 100 kb (Fig. 3). On average, LD decreased from 0.34 (0.30–0.39) to 0.25 (0.22–0.30), 0.23 (0.19–0.28), 0.16 (0.13–0.20), 0.10 (0.09–0.12) and 0.07 (0.07–0.09) for distances between markers of 10 kb, 50 kb, 100 kb, 1 Mb, 5 Mb and 10 Mb, respectively (see Additional file 1: Table S2). The unselected lines SY_n_ and SY and the commercial line SD had the lowest LD with an average *r*^2^ equal to 0.22 at 50 kb, whereas the selected lines SU, SC, SA and SB had corresponding *r*^2^ values of 0.25, 0.27, 0.27 and 0.30, respectively (see Additional file 1: Table S2).

Average *r*^2^ for the 50 kb-distant markers varied strongly between chromosomes: from 0.18 for Omy22 to 0.37 for Omy5 with an average value of 0.24 across chromosomes (see Additional file 2: Figure S1). Within chromosome, *r*^2^ varied also between lines: for Omy5, average *r*^2^ for 50 kb-distant markers varied from 0.34 for SY to 0.41 for the SU line; for Omy22, *r*^2^ varied from 0.16 for SD to 0.22 for the SA and SB lines. For some other chromosomes, in particular for the sex chromosome Omy29, variation in average *r*^2^ was more important across lines: from 0.18 for SD to 0.31 for SB.

### Estimates of effective population size

**Fig. 4:**
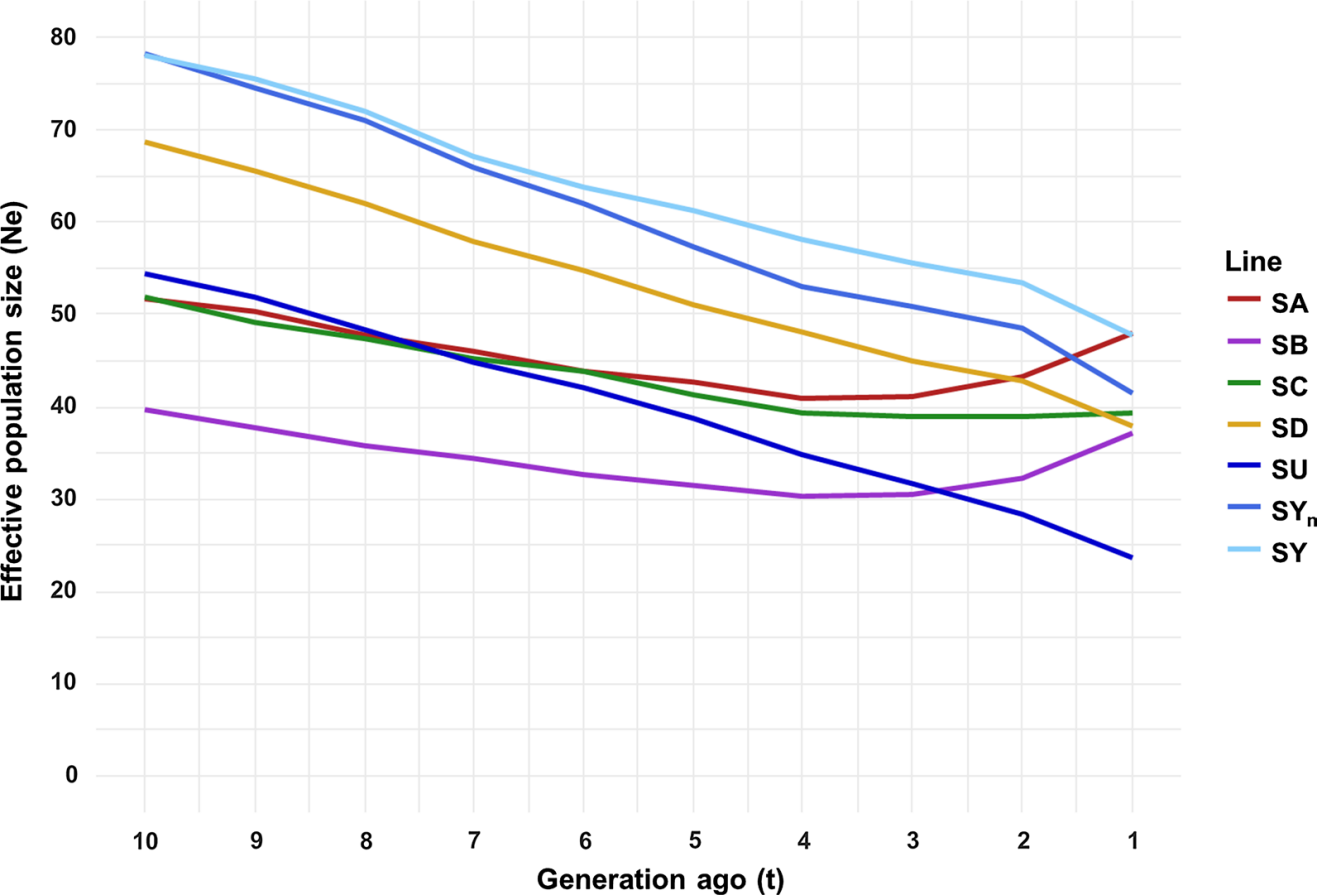
LD-based estimates of effective population size (*N*_*e*_) in the French rainbow trout lines over the last ten generations

The *N*_*e*_ of all lines showed a decreasing trend over the last 10 generations with a steeper slope for the INRA experimental lines and SD line (Fig. 4) than for the four other lines. *N*_*e*_ stabilized during the last three generations for the SA, SB and SC lines and even started to increase for the last two generations for the SA and SB lines.

In the last generation ($t = 1$), *N*_*e*_ ranged from only 24 for the SU line to 48 for the SA and SY lines, with intermediate values of 37, 38, 39 and 42 for the SB, SD, SC and SY_n_ lines, respectively. For the experimental lines SU and SY, estimates of *N*_*e*_ in the last generation ($t = 1$) due to variations in allele frequency were higher than estimates based on LD: 66 versus 24 and 216 versus 48 for the SU and SY lines, respectively (results not shown).

### Analysis of runs of homozygosity

Statistics concerning the average number and size of ROH segments per individual according to the lines are reported in Table S3 (see Additional file 1: Table S3). The average number of ROH per individual varied from 46 for the SY_n_ to 68 for the SA line. Individuals with the smallest number (25) and the largest number (90) of ROH belonged to the SY_n_ and SA lines, respectively. The average size of ROH varied from 3.84 Mb for SY_n_ to 5.38 Mb for SB. The proportion of long ROH (≥ 10 Mb) varied from 7% for SY_n_ to nearly 14% for SB.

### Estimates of total and recent inbreeding

**Fig. 5:**
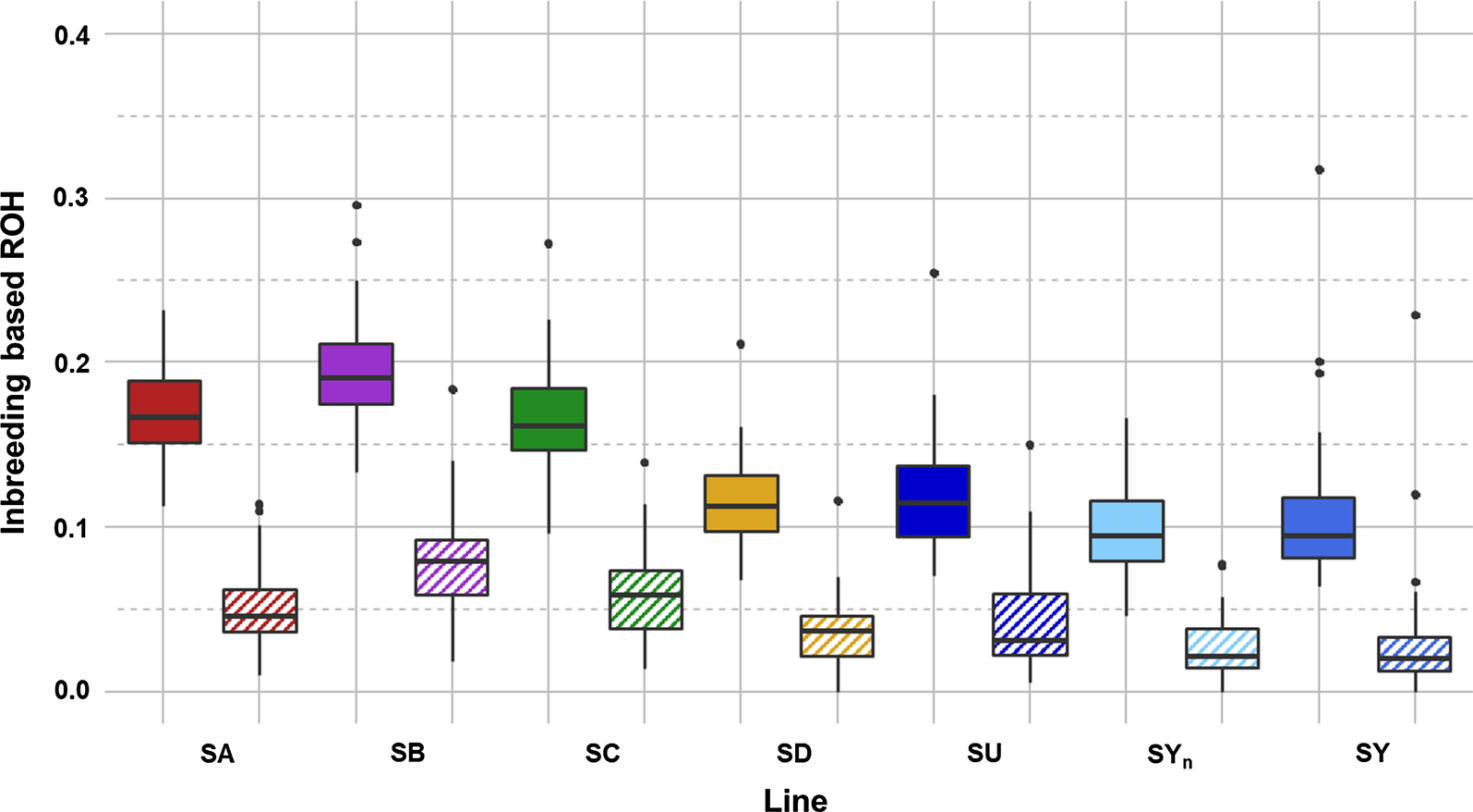
Box plots of total inbreeding (*F*_ROH_) and recent inbreeding (*F*_ROH>10_) for each rainbow trout line. Plain box: total inbreeding (*F*_ROH_); hatched box: recent inbreeding (*F*_ROH>10_)

Average *F*_ROH_ varied from 10.0% for SY_n_ to 19.5% for SB [Fig. 5 and Table S3 (see Additional file 1: Table S3)]. Individual *F*_ROH_ varied from 4.6 to 31.7% (Fig. 5). Recent inbreeding (*F*_ROH>10Mb_) ranged from 2.9 for SY_n_ to 7.9% for SB [Fig. 5 and Table S3 (see Additional file 1: Table S3)]. Individual *F*_ROH>10Mb_ varied from 0 to 22.9% for an individual in SY (Fig. 5). An increase in inbreeding of 1 point (from 10 to 11% between SY_n_ and SY) was observed in the unselected SY line in five generations. All selected lines had higher average inbreeding levels than the unselected SY line, the highest levels for total (> 16%) and recent (> 5%) inbreeding being found for the SA, SB and SC lines that are selected based on pedigree information since seven, four and six generations, respectively.

**Fig. 6:**
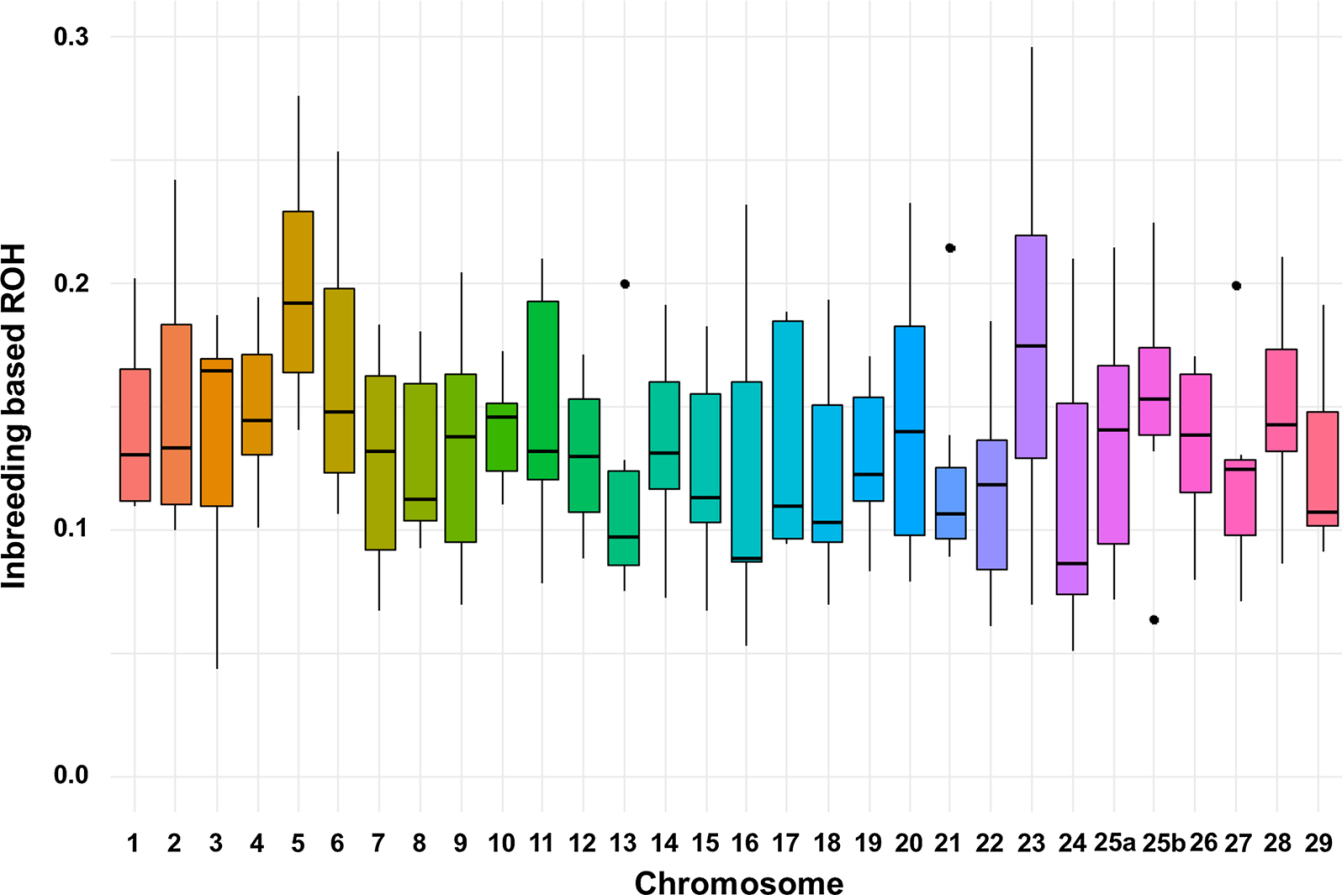
Box plots of the line averages of inbreeding coefficients (*F*_ROH_) derived from ROH per chromosome

Regarding *F*_ROH_ at the chromosome level (Fig. 6), the mean *F*_ROH_ across lines ranged from 11% for Omy13 to 20% for Omy5 with an average of 14% across chromosomes. The highest mean *F*_ROH_ (30%) was observed for SA on Omy23 and the lowest value (4%) for SY_n_ on Omy3. Average *F*_ROH_ is sometimes relatively close from one line to another (for instance for Omy10, the lowest value is 11% for SD and the highest value is 17% for SB), whereas variation in *F*_ROH_ is much larger for Omy23, from 7% for SY to 30% for SA.

## Discussion

### Population differentiation

Most of our rainbow trout populations were moderately differentiated according to the qualitative guidelines proposed by Wright [41] for the interpretation of *F*_ST_: 0–0.05 for little genetic differentiation, 0.05–0.15 for moderate genetic differentiation, 0.15–0.25 for large genetic differentiation, and above 0.25 for very large genetic differentiation, respectively.

Little genetic differentiation (*F*_ST_ < 0.03) was observed between the SU and SY experimental lines, which is reasonable given that the SU selected line was derived from the SY_n_ line. The SD commercial line was also genetically close to the SY INRA lines, partly due to the fact that about 50% of the male founders of the SD line came from the INRA sex-reversed males, 25 years ago. Although the other commercial lines are more distant from the INRA line than the SD line (Fig. 2), it is also important to note that all of these four lines were created using sex-reversed males from INRA. Among all the selected lines, *F*_ST_ values were quite similar to the observed differentiation levels between Chinese or Western pigs breeds [42], European cattle breeds [39, 43] or sheep breeds [44, 45]. As far as we know, only one *F*_ST_ study has been performed for rainbow trout based on a panel of 99 SNPs [46] and found a global F_ST_ of 0.13 across eight commercial lines and a pairwise F_ST_ between any two lines ranging from 0.056 to 0.195. Some older works based on microsatellites [47–49] or allozymes [50] also indicated a moderate degree of differentiation among wild population and/or farmed stock rainbow trout lines.

One way to assess the genetic diversity within populations is through observed (Ho) and expected (He) heterozygosity. We believe that these parameters should be derived without setting a threshold MAF value in order to keep all SNPs in the analysis and avoid any bias. In this way, we estimated an Ho of about 34% (± 1%); however, most of studies in the literature give results for a subset of SNPs with MAF above 5%. We also calculated Ho under this MAF threshold and found values close to 37% in all lines, which are very similar to the values estimated in pig lines [51] or cattle breeds [39]. Based on allozyme markers, Cárcamo et al. [50] indicated that commercial lines of rainbow trout showed a similar range of variation in heterozygosity to that of wild populations.

In our study, we observed a slight heterozygosity excess (Ho > He) with a large set of neutral loci for all lines. This observation may indicate a recent (< 100 generations) bottleneck [52] that is likely due to the recent domestication and selection process.

### Linkage disequilibrium

The LD at short distances between markers (≤ 100 kb) is very similar to *r*^2^ values reported for dairy cattle breeds [53] and slightly higher than values reported for beef cattle breeds or sheep breeds [45]; however, LD estimates for our trout lines at short distances are lower than in most pig breeds [51, 54]. At longer distances between markers (e.g. ~ 1 Mb), LD in French rainbow trout lines (0.13–0. 20) is clearly higher than in ruminant breeds, but very similar to the LD observed in most pig breeds. This high LD, even at long distances, enables accurate genomic predictions for rainbow trout populations even with low density SNP chips [55].

Similar values of *r*^2^ using long distance windows (5 Mb) were observed between the French rainbow trout lines and an American commercial line of rainbow trout [55], with a similar large variation in average *r*^2^ among chromosomes (see Additional file 2: Figure S1), i.e. very high *r*^2^ values on Omy5 and very low values on Omy21 and Omy22. The higher than average LD on Omy5 is likely caused by a large chromosomal double-inversion of 56 Mb [26], which prevents recombination in fish. This double-inversion contains key genes involved in photosensory processes, circadian rhythm, adiposity, and sexual differentiation [26]. This inversion is shown to mediate sex-specific migration through sex-dependent dominance. Quantitative trait loci (QTL) for spawning date and body weight have been detected on Omy5 [56] and may also explain the high LD because of selection of favorable haplotypes for these important traits for breeding. Furthermore, Omy5 is one major exception to the strong association observed between large metacentric chromosomes and high female:male recombination rate ratios [57]. In addition to having an equal female:male recombination rate ratio (average 9:1 ratio for metacentric chromosomes), there is evidence that the short arm of Omy5 is the homeologous linkage group to the Omy29 [58], sex chromosome for which we observed a large variation of *r*^2^ values among lines.

### Effective population size

The estimates of *N*_*e*_ for the selected lines were consistent with the reports in other aquaculture species such as the Pacific abalone [59], catfish [60], Atlantic salmon [61] for which *N*_*e*_ was less than 50 after a few generations of mass selection. The *N*_*e*_ across many livestock breeds is less than 100 [9, 51, 62]. Nevertheless, large and ongoing genetic gains for production traits are typically achieved in livestock, and there are no apparent signs of reaching selection plateaus [63]. Across both domestic and wild populations, the minimal *N*_*e*_ to avoid inbreeding depression in the short term has been estimated to be at least 50 [64]. For instance, in a long-term (120 generations) selection experiment in mice, several lines were kept at an average *N*_*e*_ of 60 with no apparent inbreeding problems [65].

In the ideal scenario, population genetics theory recommends keeping equal numbers of males and females and maintaining a constant population size over time. However in most livestock breeding programs, it is often impossible to maintain a 1:1 sex ratio, which greatly affects the *N*_*e*_ of a population. In the trout lines under study, the dams-sires ratios are relatively well balanced with values ranging from 6:10 to 10:10. In fish, an additional explanation for the small *N*_*e*_ may be asymmetric reproduction, e.g. high variance in individual reproductive success or survival rate of broodstock [66]. This last phenomenon has been described as “sweepstakes reproductive success” (SRS [67]), which maintains much less genetic diversity than expected on the basis of a large census size, and would increase the impact of inbreeding depression for aquaculture farms. Christie et al. [68] found in their review of salmon species that early-generation hatchery fish averaged only half the reproductive success of their wild-origin counterparts when spawning in the wild. For *Oncorhynchus mykiss*, the ratio of *N*_*e*_ to the estimated census population size (*N*) was estimated to range from 0.09 to 0.18 [47], 0.17 to 0.40 [69], and a 0.45 [48], with a large variance in reproductive success among individuals being the key factor to reduce the $N_{e} /N$ ratio in salmonid species [68]. Fish breeding from multigenerational hatchery program without pedigree information resulted in a decrease in *N*_*e*_, not only by decreasing the mean reproductive success but also by increasing the variance in reproductive success among breeding parents, whereas there was no reduction in *N*_*e*_ found in fish breeds for a single generation in a local hatchery [69].

Intense directional selection of relatively few animals often results in a skewed genetic contribution and may explain why *N*_*e*_ values less than 100 are observed for many livestock populations. In our study, the impact of intense selection can be quantified by comparing the evolution of *N*_*e*_ between the SU and SY lines, but also by taking into account that *N*_*e*_ is affected by the smaller number of broodstock used for SU than for SY breeding. Based on LD values, *N*_*e*_ at $t = 1$ generation was estimated to be equal to 24 and 48 for those two lines, respectively. The discrepancy was even greater when considering estimates of *N*_*e*_ based on allele frequency variation, 66 for the SU line and 216 for the SY line.

Due to the existence of two major inversions [26] on Omy5 and Omy20 that may influence our LD-based analysis (and to a lower extent the ROH), we removed these chromosomes from the derivation of *N*_*e*_ and *F*_ROH_. Values for total *F*_ROH_ and recent inbreeding were almost unchanged, but *N*_*e*_ estimates were very sensitive to the absence of Omy5 and Omy20 although *r*^2^ values were averaged per chromosome before deriving *N*_*e*_. Removing these chromosomes increases our estimates of *N*_*e*_ across all lines, on average by 40% at generation $t = 1$ and by 14% at generation $t = 10$ (see Additional file 1: Table S4). However, LD-based estimates of *N*_*e*_ remained small for all the lines compared to census sizes that were close to 200 for all commercial lines, 140 for the SY line and 80 for the SU line. Regardless of the line considered in the study (selected or not), we observed that in general *N*_*e*_ tended to decrease in the last 10 generations, which may be explained by drift and SRS phenomena. It should also be noted that in the three selected lines (SA, SB and SC) for which a mating optimization procedure to limit inbreeding was set up in the last 10 years by SYSAAF, *N*_*e*_ stabilized (SC) or even slightly increased (SA and SB) in the last three generations. This suggests that some mating optimization protocol (genomic-based pedigree) should be similarly introduced to limit the inbreeding increases in the SD, SU and SY lines.

In addition to quantifying the important decreases in *N*_*e*_ due to selection, our results underscored the difficulty in acquiring accurate estimates for this parameter, since estimates based on the same molecular information can be doubled or tripled depending on the evolutionary model considered [70]. It is well-established that better estimates of inbreeding levels and *N*_*e*_ are obtained using molecular rather than genealogical information [16], at least when considering more than 10K SNPs [71]. In most cases, a full-depth pedigree is unavailable and most base populations include selected or partially inbred founders in the pedigree files. These unknown relationships between animals may lead to strong underestimation of the pedigree-based estimates of inbreeding [16]. In addition, by considering complete pedigree back to a base population in simulation studies, Liu et al. [72] and Forutan et al. [16] reported that pedigree-based estimates of inbreeding were lower than true inbreeding in selected populations. The pedigree-based inbreeding assumes neutral loci, i.e. that the two alleles at the same locus on two homologous chromosomes have an equal chance of being selected. In reality, for some loci, the two alleles may have different effects on a naturally or artificially selected trait, which leads to unequal selection probabilities between the two alleles.

Most methods applied to infer *N*_*e*_ from genomic population data rely on the Wright–Fisher model’s assumptions of low fecundity non-skewed offspring distributions. Although proven to be robust to violations of most of these assumptions, these methods drastically failed to approximate the genealogies of species with high SRS, whereby few individuals contribute most of the offspring to the next generation [73]. As stated by Montano [70], the development of statistical tools based on models that consider SRS will substantially improve estimates of population demographic parameters.

### Runs of homozygosity

The majority of metrics to estimate *N*_*e*_ assumes that the value remains constant across the genome. However, *N*_*e*_ varies across the genome, such that some regions have an increased loss of diversity compared with others [74, 75]. The *N*_*e*_ is expected to vary across the genome as a consequence of genetic hitchhiking due to selection [76] and negative selection acting on deleterious mutations (i.e., background selection [77]). The action of selection, particularly in regions of the genome with low rates of recombination, is expected to reduce *N*_*e*_, leading to lower levels of genetic diversity and reduced effectiveness of selection. We could have derived *N*_*e*_ per chromosome based on chromosome-specific LD estimates; however, we preferred to study the heterogeneity of genetic diversity throughout the genome using ROH because *N*_*e*_ estimates have been proven unreliable for fish populations (see previous section), and quantifying the absolute level of inbreeding (in total and for each chromosome) in our rainbow trout lines was per se an objective of the study. ROH can be used to directly estimate inbreeding depression [78] and can identify the chromosome regions that are responsible for inbreeding depression along the genome [79].

While for selected rainbow trout lines, *F*_ROH_ varied between 12 and nearly 20%, estimates were a bit lower (~ 11%) for the INRA unselected line. These results are consistent with our *N*_*e*_ estimates for three to ten generations ago: the SY and SD lines had the largest *N*_*e*_ and the lowest *F*_ROH_ whereas SB had the smallest *N*_*e*_ and the highest *F*_ROH_ (Pearson correlation *F*_ROH_ and $N_{{e_{t = 10} }}$: − 0.90, p value < 0.005). It is difficult to compare our results to other broodstock programs because the few published estimations of levels of inbreeding for fish commercial populations are all based on pedigree information, and thus are likely to be underestimated due to unknown relationships and inbreeding in the base populations. In rainbow trout, Pante et al. [80] estimated inbreeding levels that varied between 3 and 10% at the 6th generation of selection for three commercial populations. A hierarchical mating system was used (one male mated to two to three females) with a very variable number of families from one generation to another; full-sib and half-sib matings were avoided and matings that would yield inbreeding coefficients of 12.5% or more were restricted. In Coho salmon, Myers et al. [81] reported inbreeding levels of about 15–16% in two lines after the 9th and 10 generations of the Domsea selection program based on a circular mating design with 60 families produced at each generation [82]. More recently, in two different lines of Coho salmon, Yáñez et al. [83] estimated inbreeding levels at 5 and 7% after 6 and 7 generations of selection, respectively, under a hierarchical design (one male mated to three to five females) with 100 families produced at each generation and inbreeding controlled by avoiding half- and full-sib matings.

While empirical information on genome-wide diversity in model species and livestock has been collected, we still lack a clear picture for farmed fish of the heterogeneity of genetic diversity across the genome. As far as we know, our study is the first to calculate ROH and estimate *F*_ROH_ in any fish species. *F*_ROH_ estimates from this study were higher than estimates for terrestrial livestock regardless of their breeding management (in breeds for ruminants or in lines for pigs). Estimates varied from 3 to 9% in dairy cattle breeds [15, 38, 39], from 2 to 11% in sheep breeds [84, 85] and from 3 to 11% in pig lines [51, 86, 87].

Although the SY line is not under selective breeding, inbreeding is relatively high (11%) and has increased by 1% in six generations (see Additional file 1: Table S3). This is probably partly due to a founder effect as well as to SRS, as previously discussed for *N*_*e*_. In spite of the existence of mating plans for selected lines with known pedigree (SA, SB and SC), recent inbreeding is relatively high in these lines (5–8%). Nevertheless, we may be able to manage their genetic diversity along the entire genome. Because no ROH fragments are fixed within the populations (see Additional file 2: Figure S2), we can develop selection and mating strategies to specifically limit inbreeding in genome regions for which an important proportion of individuals (> 50% for instance) share the same ROH.

It is worthwhile to underscore that *F*_ROH_ estimates may vary depending on the parameters considered to define ROH segments. These parameters have to be tuned according to marker density and pan-genomic heterogeneity and the level of recombination along the genome. There is no clear consensus in the literature on how to choose these parameters and most studies used Plink default values. In a recent study [16], a gene-dropping simulation was performed and inbreeding estimates based on ROH and pedigree data were compared to true inbreeding. Inbreeding based on ROH was estimated using different software and different threshold parameters using 50 K chip data. While pedigree inbreeding underestimated true inbreeding, using ROH with a minimum window size of 20 to 50 SNPs provided the closest estimates to true inbreeding regardless of the software [16, 88]. In our study, we observed that inbreeding estimates were similar for window sizes of 30 and 50 SNPs, and estimates were almost identical when considering recent inbreeding (results not shown). Nevertheless the results were sensitive to the maximum gap between two successive SNPs (1000 kb vs. 250 kb) and, to a lesser extent, to the MAF threshold (see Additional file 1: Table S3). Due to the heterogeneity of the marker density along the genome, long ROH were fragmented into smaller ones when considering a maximum gap of 250 kb, leading to underestimated *F*_ROH_ in our lines. The largest differences were for recent inbreeding estimates, *F*_ROH≥10_, that were less than 1% for the gap threshold of 250 kb, but varied between 3 and almost 8% for the 1000 kb threshold (see Additional file 1: Table S3).

As shown in previous studies on the sheep genome [45, 85, 89], we observed a strong variation in *F*_ROH_ values among chromosomes, these results being consistent with variation in LD among chromosomes. Chromosome ends are less represented in ROH segments (results not shown), probably due to the very low marker density in these areas. Hotspot areas for ROH were observed for each chromosome (see Additional file 2: Figure S2). In general, these areas and the proportions of individuals within a population that shared ROH hotspots in those areas were highly variable from one line to another. Nevertheless a few hotspot areas were commonly detected across lines, such as the position at ~ 48 Mb of Omy10 (see Additional file 2: Figure S3). At this specific position, is located a QTL for bacterial cold disease resistance that was detected in an American rainbow trout population [90] and in a French commercial line [91]. When hotspot areas are common to different lines, they may be due to local low recombination rates [85] or to key genes for domestication or production [39, 40, 85]. These hotspot areas could then help identify important genes for domestication or production.

The accumulation of inbreeding is heterogeneous across the genome, such that certain regions are being inbred at a faster rate than other regions of the genome. Currently, SNP information is predominantly used to predict breeding values for genomic selection in livestock populations, now including farmed fish (catfish, tilapia and salmonids). However, this information could also be used to manage the rate at which inbreeding builds up in the genome of livestock populations. Characterizing and efficiently managing inbreeding levels is a major goal to ensure that populations can adapt to future breeding goals while maintaining genetic diversity and avoiding the accumulation of detrimental effects associated with inbreeding. Identifying the main regions of the genome that contribute to inbreeding depression may help to manage inbreeding not only at the individual level but directly at the genome level.

## Conclusions

Our findings provide the breeding companies a better understanding of the genetic diversity in their rainbow trout lines in order to implement efficient breeding programs. The availability of a genome-wide SNP chip allowed us to characterize genetic diversity between and within lines. Lines are moderately differentiated across commercially developed trout lines, with similar *F*_ST_ values as those reported across ruminant breeds or pig lines. Within each line, effective population size seems rather small and inbreeding levels are higher than in terrestrial livestock selected populations. This may be explained by founder effects, sweepstakes reproductive success, and intense selection in some lines. The impact of these significant levels of inbreeding on rainbow trout performance should be quantified in order to assess potential inbreeding depression phenomena and risks for future genetic gains. The levels of molecular inbreeding derived from the identification of homozygous genomic segments could also be used to directly identify the regions that are responsible for inbreeding depression along the genome. We could then expect a more efficient purging allowing for higher relatedness between selected individuals without inbreeding drawbacks in breeding programs.

## Additional files

**Additional file 1.** **Table S1**: Number of SNPs per minimum allele frequency (MAF) category in each line. **Table S2**: Average *r*^2^ ± SD between SNPs according to different distances. **Table S3**: Sensitivity analysis of ROH estimates to MAF and maximal distance gap between two SNPs and derived inbreeding coefficient. **Table S4**: Estimates of effective population size (standard errors in brackets) for each line with or without Omy5 and Omy20.

**Additional file 2.** **Figure S1**: Line mean linkage disequilibrium at 50 kb for each chromosome. **Figure S2**: Proportion of individuals per line with a SNP in a ROH along the genome. **Figure S3**: Proportion of individuals per line with a SNP in a ROH along chromosome 10.

## Data Availability

The datasets generated and/or analyzed during the current study are not publicly available because they belong to commercial breeding companies but are available from the corresponding author on reasonable request and with permission of the relevant companies.
