# Supplementary material for: Genome-wide estimates of genetic diversity, inbreeding and effective size of experimental and commercial rainbow trout lines undergoing selective breeding
Source: Genet Sel Evol. 2019 Jun 6;51:26. doi: 10.1186/s12711-019-0468-4 (PMC6554922; doi:10.1186/s12711-019-0468-4)
Supplement: Supplementary file 1 — Additional file 1. Table S1: Number of SNPs per minimum allele frequency (MAF) category in each line. Table S2: Average r2 ± SD between SNPs according to different distances. Table S3: Sensitivity analysis of ROH estimates to MAF and maximal distance gap between two SNPs and derived inbreeding coefficient. Table S4: Estimates of effective population size (standard errors in brackets) for each line with or without Omy5 and Omy20. [file 12711_2019_468_MOESM1_ESM.docx]

**Table S1.** Number of SNP per minimum allele frequency (MAF) category in each line.

| **Line** | **MAF < 1%** | **1% ≤MAF <5%** | **MAF ≥5%** | **MAF ≥ 25%** |
| --- | --- | --- | --- | --- |
| SA | 2,884 | 3,432 | 32,034 | 16,263 |
| SB | 4,273 | 2,887 | 31,190 | 16,716 |
| SC | 3,162 | 3,286 | 31,902 | 16,915 |
| SD | 1,363 | 2,810 | 34,175 | 18,726 |
| SU | 2,132 | 3,025 | 33,201 | 17,865 |
| SY_n_ | 1,010 | 2,622 | 34,723 | 19,173 |
| SY | 1,389 | 2,621 | 34,341 | 19,173 |

**Table S2.** Average r² ± SD between SNPs according to different distances.

| **Distance (Kb)** | **SA** | **SB** | **SC** | **SD** | **SU** | **SY_n_** | **SY** |
| --- | --- | --- | --- | --- | --- | --- | --- |
| 10 | 0.35 ± 0.34 | 0.39 ± 0.36 | 0.36 ± 0.35 | 0.31 ± 0.32 | 0.34 ± 0.33 | 0.30 ± 0.32 | 0.31 ± 0.32 |
| 50 | 0.27 ± 0.29 | 0.30 ± 0.31 | 0.27 ± 0.29 | 0.22 ± 0.26 | 0.25 ± 0.27 | 0.22 ± 0.25 | 0.22 ± 0.25 |
| 100 | 0.25 ± 0.27 | 0.28 ± 0.30 | 0.25 ± 0.27 | 0.20 ± 0.24 | 0.22 ± 0.25 | 0.19 ± 0.23 | 0.20 ± 0.23 |
| 1000 | 0.17 ± 0.21 | 0.20 ± 0.24 | 0.17 ± 0.21 | 0.14 ± 0.18 | 0.16 ± 0.21 | 0.13 ± 0.18 | 0.13 ± 0.18 |
| 3000 | 0.12 ± 0.17 | 0.14 ± 0.19 | 0.12 ± 0.17 | 0.10 ± 0.15 | 0.13 ± 0.18 | 0.10 ± 0.15 | 0.10 ± 0.15 |
| 5000 | 0.10 ± 0.16 | 0.12 ± 0.18 | 0.10 ± 0.16 | 0.09 ± 0.14 | 0.11 ± 0.17 | 0.09 ± 0.15 | 0.09 ± 0.14 |
| 10000 | 0.07 ± 0.13 | 0.08 ± 0.14 | 0.07 ± 0.14 | 0.07 ± 0.13 | 0.09 ± 0.15 | 0.07 ± 0.13 | 0.07 ± 0.12 |
| 30000 | 0.04 ± 0.11 | 0.05 ± 0.11 | 0.05 ± 0.12 | 0.05 ± 0.10 | 0.07 ± 0.13 | 0.06 ± 0.11 | 0.05 ± 0.10 |

**Table S3**. Sensitivity analysis of ROH estimates to MAF and maximal distance gap between two SNPs and derived inbreeding coefficient.

| **ROH with (MAF1%, max gap 1000kb)** | | | | | | |
| --- | --- | --- | --- | --- | --- | --- |
| **Line** | **Number of segments** | **Number of SNPs per segment** | **Size of segments (Mb)** | **% ROH>10Mb** | **F_ROH_** | **F_ROH>10Mb_** |
| SA | 68.2 (± 9.4) | 96 (± 91) | 4.51 (± 4.19) | 8.86 | 0.171 (± 0.027) | 0.051 (± 0.024) |
| SB | 65.2 (± 7.3) | 110 (± 107) | 5.38 (± 5.21) | 13.57 | 0.195 (± 0.034) | 0.079 (± 0.032) |
| SC | 65 (± 9.7) | 98 (± 100) | 4.60 (± 4.71) | 10.08 | 0.166 (± 0.034) | 0.058 (± 0.027) |
| SD | 50.2 (± 9) | 93 (± 99) | 4.14 (± 4.42) | 7.67 | 0.116 (± 0.027) | 0.036 (± 0.020) |
| SU | 49.9 (± 9.6) | 97 (± 114) | 4.40 (± 5.24) | 9.33 | 0.122 (± 0.040) | 0.045 (± 0.035) |
| SY_n_ | 46.2 (± 10.5) | 87 (± 87) | 3.84 (± 3.97) | 6.96 | 0.100 (± 0.030) | 0.029 (± 0.019) |
| SY | 49.7 (± 9.5) | 89 (± 98) | 4.01 (± 4.52) | 7.32 | 0.112 (± 0.049) | 0.034 (± 0.042) |
| **ROH with (MAF 1%, max gap 250kb)** | | | | | | |
| **Line** | **Number of segments** | **Number of SNPs per segment** | **Size of segments (Mb)** | **% ROH>10Mb** | **F_ROH_** | **F_ROH>10Mb_** |
| SA | 77.9 (± 11.1) | 75 (± 57) | 3.15 (± 2.32) | 1.82 | 0.160 (± 0.026) | 0.013 (± 0.011) |
| SB | 80.6 (± 10.9) | 78 (± 57) | 3.30 (± 2.33) | 2.51 | 0.176 (± 0.032) | 0.016 (± 0.013) |
| SC | 76.7 (± 12.5) | 75 (± 56) | 3.11 (± 2.29) | 2.05 | 0.156 (± 0.032) | 0.013 (± 0.011) |
| SD | 56.1 (± 11) | 76 (± 63) | 3.04 (± 2.45) | 2.53 | 0.110 (± 0.026) | 0.013 (± 0.012) |
| SU | 56.6 (± 14.1) | 77 (± 67) | 3.14 (± 2.69) | 3.04 | 0.115 (± 0.040) | 0.015 (± 0.013) |
| SY_n_ | 49.5 (± 12.4) | 73 (± 59) | 2.89 (± 2.28) | 1.77 | 0.092 (± 0.030) | 0.008 (± 0.010) |
| SY | 53.5 (± 15) | 74 (± 63) | 2.98 (± 2.45) | 2.66 | 0.103 (± 0.047) | 0.013 (± 0.019) |
| **ROH with (MAF 5%, max gap 1000kb)** | | | | | | |
| **Line** | **Number of segments** | **Number of SNPs per segment** | **Size of segments (Mb)** | **% ROH>10Mb** | **F_ROH_** | **F_ROH>10Mb_** |
| SA | 61 (± 8.8) | 93 (± 85) | 4.8 (± 4.37) | 9.57 | 0.163 (± 0.027) | 0.050 (± 0.023) |
| SB | 58.1 (± 7.5) | 108 (± 101) | 5.75 (± 5.43) | 15.20 | 0.186 (± 0.034) | 0.079 (± 0.032) |
| SC | 58 (± 8.9) | 96 (± 95) | 4.91 (± 4.85) | 11.29 | 0.159 (± 0.034) | 0.058 (± 0.027) |
| SD | 46.4 (± 8.6) | 91 (± 94) | 4.35 (± 4.55) | 8.31 | 0.112 (± 0.027) | 0.035 (± 0.020) |
| SU | 45 (± 9.2) | 95 (± 108) | 4.69 (± 5.46) | 10.35 | 0.117 (± 0.039) | 0.045 (± 0.035) |
| SY_n_ | 42.1 (± 9.7) | 86 (± 84) | 4.03 (± 4.17) | 7.94 | 0.094 (± 0.029) | 0.029 (± 0.020) |
| SY | 45.1 (± 9.3) | 88 (± 94) | 4.24 (± 4.65) | 8.13 | 0.106 (± 0.049) | 0.033 (± 0.042) |
| **ROH with (MAF 5%, max gap 250kb)** | | | | | | |
| **Line** | **Number of segments** | **Number of SNPs per segment** | **Size of segments (Mb)** | **% ROH>10Mb** | **F_ROH_** | **F_ROH>10Mb_** |
| SA | 71.4 (± 11.2) | 68 (± 46) | 3.06 (± 1.94) | 1.17 | 0.147 (± 0.025) | 0.007 (± 0.008) |
| SB | 74.1 (± 11.3) | 70 (± 47) | 3.16 (± 2.02) | 1.40 | 0.160 (± 0.031) | 0.008 (± 0.009) |
| SC | 68.8 (± 12.5) | 68 (± 49) | 3.00 (± 2.03) | 1.36 | 0.140 (± 0.031) | 0.008 (± 0.008) |
| SD | 52.5 (± 10.7) | 71 (± 56) | 3.04 (± 2.29) | 2.06 | 0.105 (± 0.025) | 0.010 (± 0.009) |
| SU | 52.2 (± 14.1) | 71 (± 59) | 3.07 (± 2.49) | 2.33 | 0.107 (± 0.038) | 0.011 (± 0.011) |
| SY_n_ | 45.4 (± 12.3) | 70 (± 52) | 2.89 (± 2.13) | 1.45 | 0.086 (± 0.030) | 0.006 (± 0.008) |
| SY | 48.7 (± 15.1) | 71 (± 54) | 2.99 (± 2.21) | 2.61 | 0.096 (± 0.045) | 0.010 (± 0.015) |

**Table S4.** Estimates of effective population size (standard errors in brackets) for each line derived with or without Omy5 and Omy20 LD values.

|  | **Ne based on all chromosomes** | | | **Ne ignoring Omy5 and Omy20** | | |
| --- | --- | --- | --- | --- | --- | --- |
| **Generation**  **Line** | **-1** | **-5** | **-10** | **-1** | **-5** | **-10** |
| **SA** | 48 (1.1) | 43 (0.7) | 52 (1.3) | 68 (2.1) | 51 (0.8) | 58 (1.5) |
| **SB** | 37 (0.7) | 31 (0.4) | 40 (0.9) | 49 (1.2) | 35 (0.5) | 43 (1.1) |
| **SC** | 39 (0.6) | 41 (0.6) | 52 (1.2) | 61 (1.5) | 49 (0.7) | 58 (1.5) |
| **SD** | 38 (0.6) | 51 (0.7) | 69 (1.6) | 54 (1.2) | 62 (1.0) | 79 (2.0) |
| **SU** | 24 (0.4) | 39 (0.6) | 54 (1.4) | 32 (0.7) | 47 (0.8) | 62 (1.7) |
| **SY_n_** | 42 (0.9) | 57 (1.0) | 78 (2.2) | 61 (2.0) | 73 (1.4) | 93 (2.8) |
| **SY** | 48 (2.2) | 31 (1.3) | 78 (2.5) | 63 (2.1) | 77 (1.5) | 91 (2.7) |
