# Supplementary material for: Genome-wide estimates of genetic diversity, inbreeding and effective size of experimental and commercial rainbow trout lines undergoing selective breeding
Source: Genet Sel Evol. 2019 Jun 6;51:26. doi: 10.1186/s12711-019-0468-4 (PMC6554922; doi:10.1186/s12711-019-0468-4)

**Fig. S1**. Line mean linkage disequilibrium at 50 Kb for each chromosome.


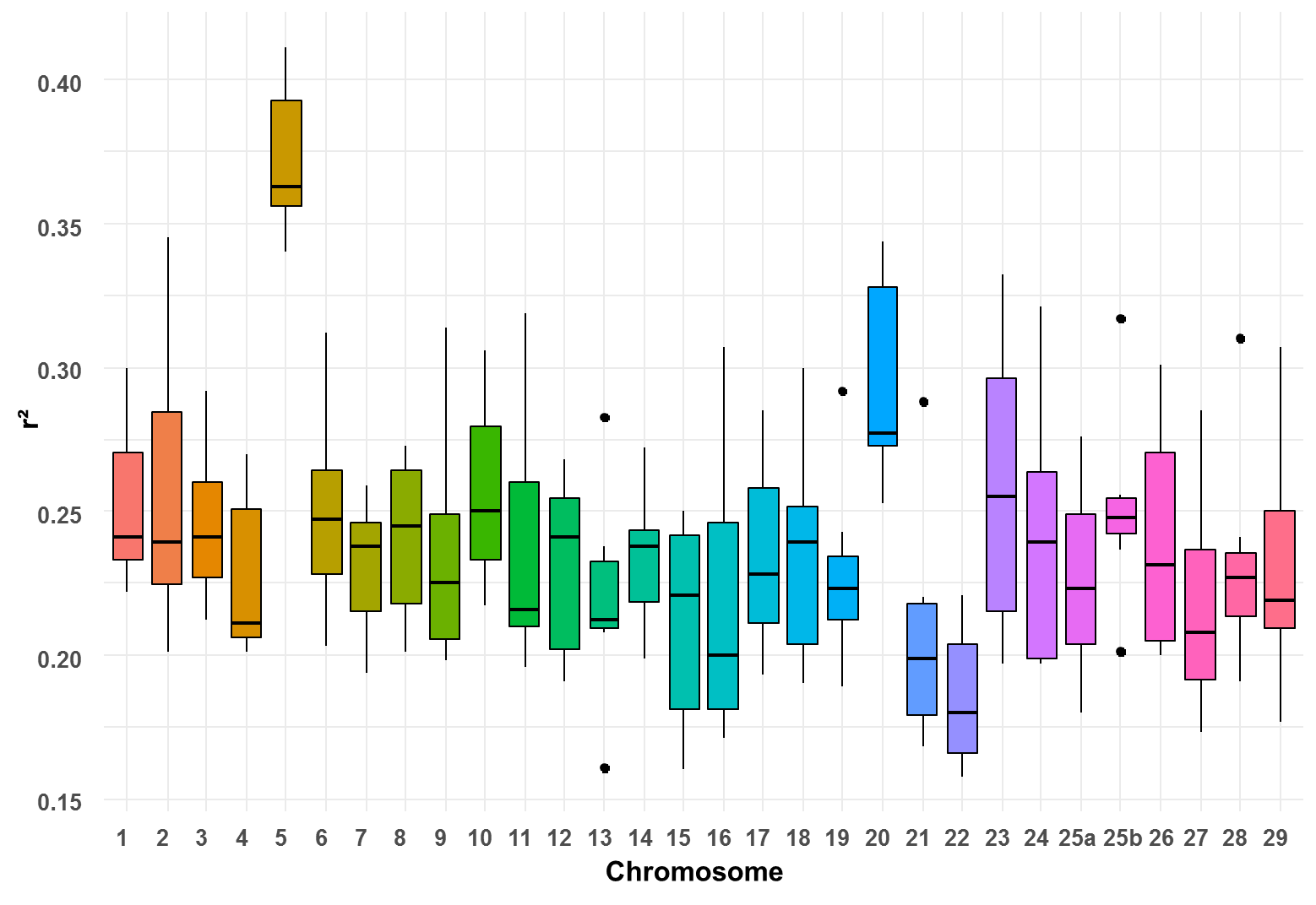


SC

SD

SU

SYn_+5_

**Fig. S2.** Proportion of individuals per line with a SNP in a ROH along the genome.


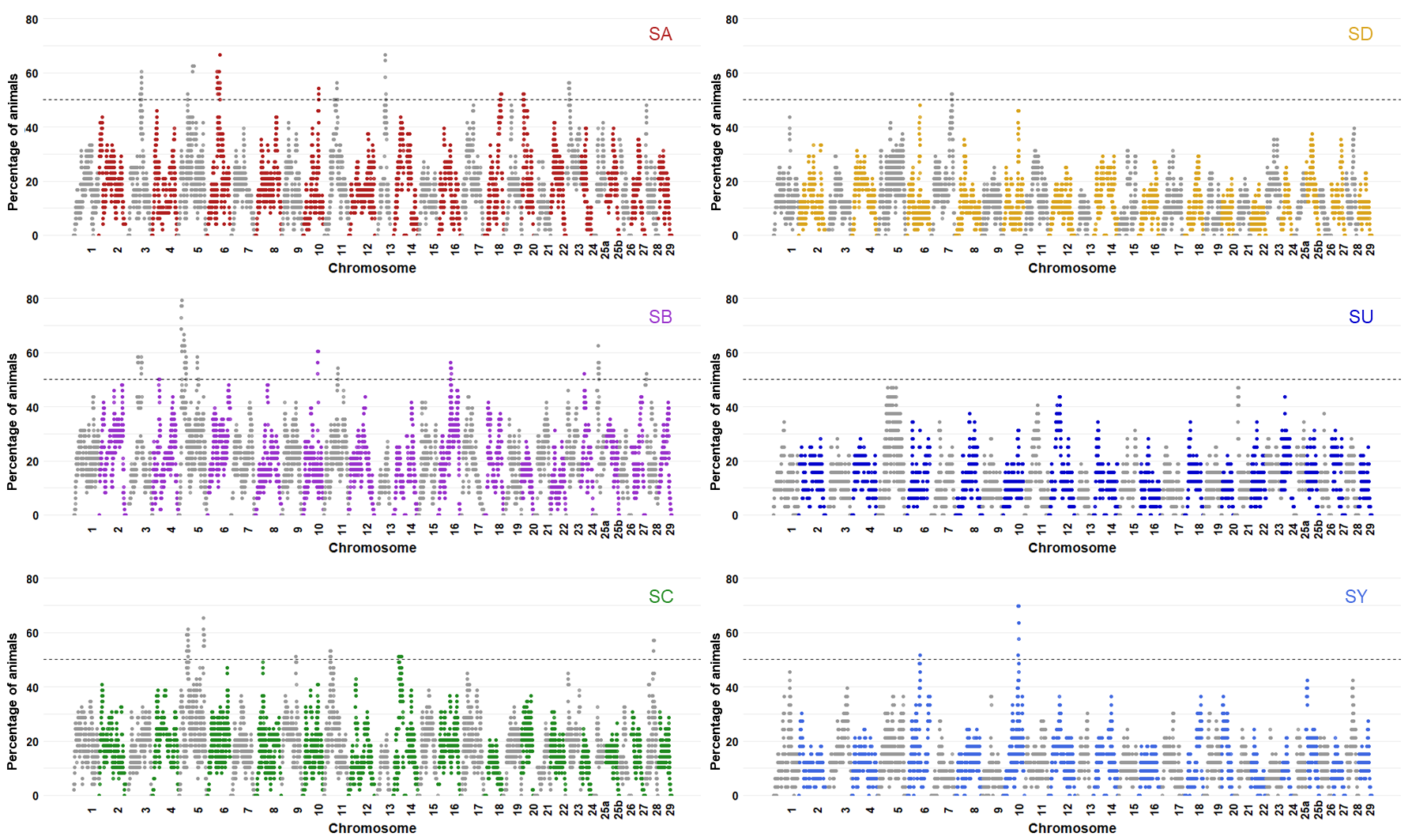


**Figure S3**. Proportion of individuals per line with a SNP in a ROH along the chromosome 10.


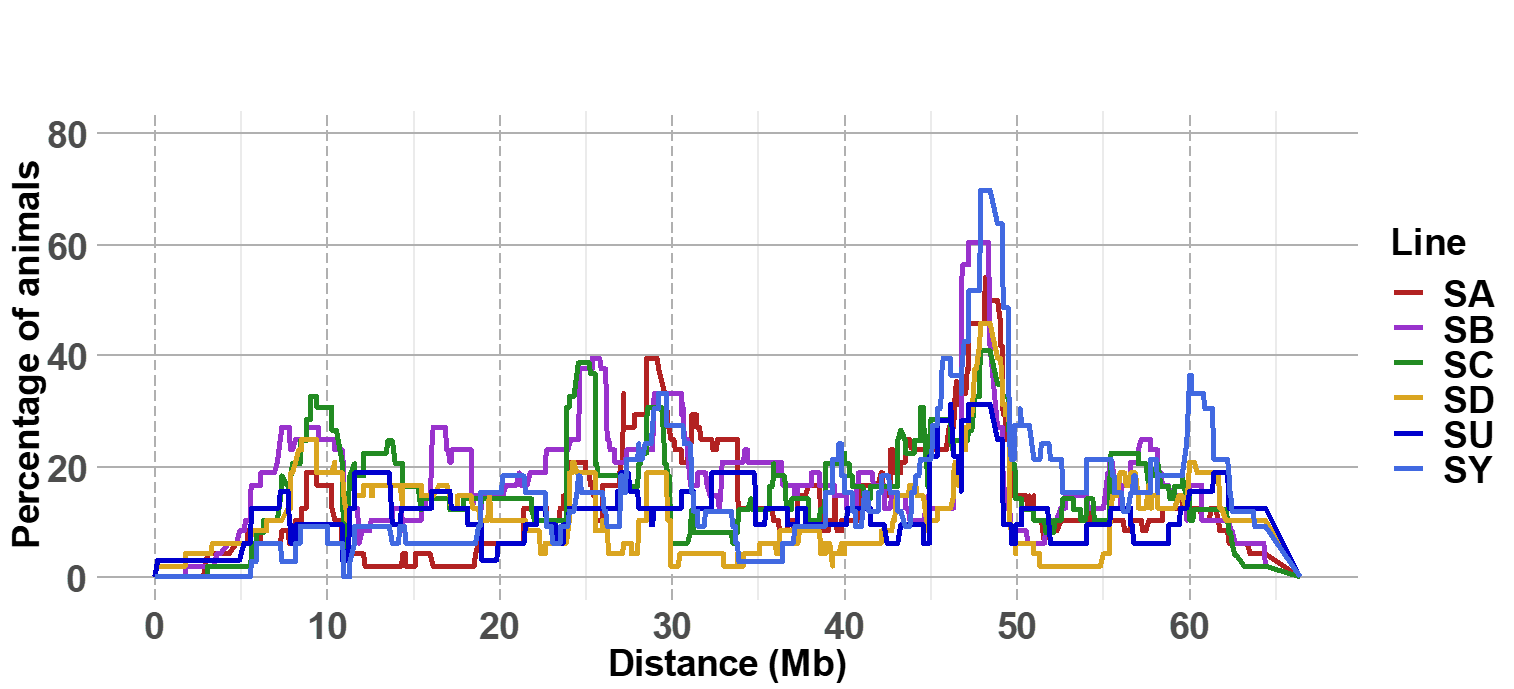

Supplement: Supplementary file 2 — Additional file 2. Figure S1: Line mean linkage disequilibrium at 50 kb for each chromosome. Figure S2: Proportion of individuals per line with a SNP in a ROH along the genome. Figure S3: Proportion of individuals per line with a SNP in a ROH along chromosome 10. [file 12711_2019_468_MOESM2_ESM.docx]
